# O2.5. MULTISENSORY INTEGRATION UNDERLYING BODY OWNERSHIP IN SCHIZOPHRENIA AND INDIVIDUALS AT FAMILIAL RISK TO DEVELOP PSYCHOSIS: A STUDY USING THE RUBBER HAND ILLUSION PARADIGM

**DOI:** 10.1093/schbul/sby015.195

**Published:** 2018-04-01

**Authors:** Neeltje Van Haren, Merel Prikken, Anouk van der Weiden, Heleen Baalbergen, Manon Hillegers, Henk Aarts, René Kahn

**Affiliations:** 1 University Medical Centre Utrecht, Brain Centre Rudolf Magnus; 2 Utrecht University, Social and Behavioural Sciences; 3 Erasmus MC, Rotterdam; 4 Icahn School of Medicine at Mount Sinai

## Abstract

**Background:**

Patients with schizophrenia suffer from fundamental self-disturbances and have difficulties integrating and distinguishing between the self and others. For example, they experience that bodily boundaries vanish, that body parts are located at the wrong part of the body or that they are not the subject of their own movements. Such experiences are referred to as disturbances in the sense of body ownership. Although these are well-described psychotic symptoms, surprisingly little is known about their etiology and development. Our aim was to replicate a more flexible sense of body ownership in patients, thereby using a well-controlled experimental procedure (with proprioceptive drift and subjective strength of the illusion. Second, we examine whether increased familial risk to develop psychosis (i.e., offspring of patients with schizophrenia), relative to increased familial risk to develop mood disorders or the absence of familial risk, is related to alterations in RHI measures.

**Methods:**

With a Rubber Hand Illusion (RHI) paradigm, body ownership was assessed in two different cohorts: 1) 54 patients with schizophrenia and 56 age and gender matched controls and 2) 24 children/adolescents with at least one parent with schizophrenia, 33 children/adolescents with at least one parent with bipolar disorder, and 18 age and gender matched controls. In this paradigm, a visible rubber hand and the invisible real hand were stroked either synchronously or asynchronously. Subsequently, proprioceptive drift and subjective RHI were measured.

**Results:**

All groups showed the rubber hand illusion, i.e., a stronger proprioceptive drift and higher subjective ratings of the RHI after synchronous compared with asynchronous stroking (all p<0.001). The effect of synchronicity on subjective RHI was significantly stronger in patients with schizophrenia as compared with healthy individuals (p=0.03). No significant differences were found between children/adolescents with and without increased familial risk to develop psychosis. Last, in patients the subjective RHI was related to severity of delusions (rho=0.36).

**Discussion:**

This study confirms alterations in embodied ownership experiences in patients with schizophrenia, but no evidence was found for impairments in children/adolescence with increased familial or clinical risk to develop psychosis. Longitudinal data are needed to reveal whether impairments in body ownership are predictive of psychosis onset, however, our findings provide suggestive evidence that this is not the case. In addition, that group differences were found in multisensory integration processes related to the embodiment, but not proprioceptive drift, implicates different underlying mechanisms. A possible explanation might come from the distinction between bottom-up (i.e., sensory input) and top-down (i.e., cognitive representation of body schema) mechanisms that influence multisensory integration, that is, altered cognitive representations may influence embodiment but not proprioceptive drift.

